# Unifying the analysis of high-throughput sequencing datasets: characterizing RNA-seq, 16S rRNA gene sequencing and selective growth experiments by compositional data analysis

**DOI:** 10.1186/2049-2618-2-15

**Published:** 2014-05-05

**Authors:** Andrew D Fernandes, Jennifer NS Reid, Jean M Macklaim, Thomas A McMurrough, David R Edgell, Gregory B Gloor

**Affiliations:** 1, YouKaryote Genomics, London, ON, Canada; 2 Department of Biochemistry, Medical Science Building, University of Western Ontario, 1151 Richmond St, N6A 5C1, London, ON, Canada

**Keywords:** compositional data, differential abundance, centered log-ratio transformation, Dirichlet distribution, Monte Carlo sampling, RNA-seq, microbiome, 16S rRNA gene sequencing, high-throughput sequencing

## Abstract

**Background:**

Experimental designs that take advantage of high-throughput sequencing to generate datasets include RNA sequencing (RNA-seq), chromatin immunoprecipitation sequencing (ChIP-seq), sequencing of 16S rRNA gene fragments, metagenomic analysis and selective growth experiments. In each case the underlying data are similar and are composed of counts of sequencing reads mapped to a large number of features in each sample. Despite this underlying similarity, the data analysis methods used for these experimental designs are all different, and do not translate across experiments. Alternative methods have been developed in the physical and geological sciences that treat similar data as compositions. Compositional data analysis methods transform the data to relative abundances with the result that the analyses are more robust and reproducible.

**Results:**

Data from an in vitro selective growth experiment, an RNA-seq experiment and the Human Microbiome Project 16S rRNA gene abundance dataset were examined by ALDEx2, a compositional data analysis tool that uses Bayesian methods to infer technical and statistical error. The ALDEx2 approach is shown to be suitable for all three types of data: it correctly identifies both the direction and differential abundance of features in the differential growth experiment, it identifies a substantially similar set of differentially expressed genes in the RNA-seq dataset as the leading tools and it identifies as differential the taxa that distinguish the tongue dorsum and buccal mucosa in the Human Microbiome Project dataset. The design of ALDEx2 reduces the number of false positive identifications that result from datasets composed of many features in few samples.

**Conclusion:**

Statistical analysis of high-throughput sequencing datasets composed of per feature counts showed that the ALDEx2 R package is a simple and robust tool, which can be applied to RNA-seq, 16S rRNA gene sequencing and differential growth datasets, and by extension to other techniques that use a similar approach.

## Background

The objective of many high-throughput sequencing studies is to identify those genes or features that make a significant difference between two or more conditions. These methods are diverse and include RNA sequencing (RNA-seq), chromatin immunoprecipitation sequencing (ChIP-seq), and metagenomic and 16S rRNA gene amplification analysis of microbial populations. All of these study designs share common aspects whereby DNA fragments are incorporated into a library, a small proportion of that library is sequenced on an instrument and the reads from the sequencing run are binned into features that represent an underlying biological entity. The entities can be genes, other expressed or non-expressed genomic features (RNA-seq and metagenomics), operational taxonomic units (OTUs) (16S rRNA gene sequencing) or genomic segments (ChIP-seq). Different statistical models are used to determine differential abundance in each type of study despite the underlying similarity in study design. The RNA-seq field has largely standardized on estimating the variation with the negative binomial [1] and a relatively small number of normalization methods [2]. In contrast, the standard 16S tag-sequencing workflow normalizes abundances between samples by rarefaction or other subsampling methods and usually works with proportions [3,4], although some suggest that these normalizations make little difference to the outcome [5]. Some groups additionally use quantitative PCR approaches to normalize reads [6]. Finally, ChIP-seq analyses often use Poisson-based models [7]. The methodological diversity appears to be due, in part, to historical accident as each field developed methods that derived from its own field of study and then became crystallized. The result is that methods used for one experimental design are often not suitable for another. However, all these tools treat the underlying data as counts per feature, adjusting these counts across samples and performing statistical tests on these adjusted counts [1,3,4,7]. Tools designed for one study design generally fail when applied to another (e.g., see [8,9]): this unexpected fragility suggests that the tools have been optimized to give biologically plausible results by latent parameterization.

### Compositional data and high-throughput sequencing

Despite current advice to treat high-throughput sequencing datasets as count data [1,10], fundamentally, such datasets are compositional [8,9,11]. That is, the total number of reads obtained for a particular sample is not itself informative. A dataset is compositional when the sum of the values for each sample is predefined [12]. Datasets of this type can be proportional, such as fractions of the whole, percentages, parts per million, etc. It is simplest to think of compositional values as being proportions of a unit, varying between 0 and 1. Proportional datasets are very different from datasets composed of ordinary numbers that can take any value.

Treating high-throughput sequencing datasets as compositions is intuitive if one considers that the primary determinant of the observed sequencing depth is the sequencing platform used and the number of samples that are multiplexed per run. For example, an Illumina HiSeq can currently generate over 200 million reads per lane, a MiSeq approximately 20 million reads per run and an Ion Torrent instrument up to 6 million reads per run. Setting aside confounding effects such as the accuracy of the instrument, clearly the direct comparison of different numbers of reads per category would be erroneous. Thus, we must think of datasets derived from high-throughput sequencing as compositions instead of count data. The purpose of this work is to show that datasets from a wide variety of experimental designs, including RNA-seq, 16S rRNA gene sequencing and selective growth (selex) experiments share a similar underlying structure, and can be analyzed appropriately using methods developed and used for decades in fields as diverse as geology, ecology and paleontology [12-15].

There have been many warnings regarding the use of standard statistical methods that assume the independence of the underlying observations when examining compositional data, the first being given by Karl Pearson in 1896 [16]. These warnings were ignored initially because there were no alternative methods. However, beginning in the late 1970s and continuing to this day, a number of approaches have been developed that fully use multivariate statistical approaches to examine compositional differences between samples. In 1986, Aitchison [12] presented a full set of rationales and detailed descriptions of what follows.

The major problem with compositional datasets is that the data points do not map to Euclidean space, but instead to a hyperplane referred to as the Aitchison simplex [12]. Aitchison demonstrated that data mapping to the simplex must be transformed prior to analysis to prevent erroneous conclusions [12,17]. The appropriateness of a data transformation for compositional data can be addressed by answering two questions about the data [17]. First, is the total sum of the counts of the data useful? And second, is the absolute difference between observations important? Answering yes to both means that the data belongs to Euclidean space, and so traditional statistical methods are valid. Answering no to both means that the data belongs to the Aitchison simplex, and it must be transformed prior to analysis. Note that by not answering these questions the investigator is assuming that the values in the dataset are count data and that the absolute difference between values is important: i.e., the investigator is assuming the values are Euclidean. This is the assumption made by all RNA-seq analysis tools, the major tools used for 16S rRNA gene analysis (mothur, qiime and VEGAN) and tools to analyze ChIP-seq.

Compositional datasets present several special challenges. The first challenge is that compositional data must be analyzed in a scale-invariant way: that is, the answer should be the same whether the investigator is dealing with proportions, percentages, ppm or per feature values where the total value is constrained to be the sum of the parts [12]. Compositional data are also dimensionless since they are proportions where the numerator and denominator have the same units. That data generated by high-throughput sequencing approaches must be analyzed in a scale-invariant manner is implied by the various corrections for read depth used by the RNA-seq community [2], and by the rarefaction or jack-knifing commonly used by common 16S rRNA gene analysis tools [18]. The second challenge is that the count values for features in a sample are not independent. In these datasets the value of one feature necessarily restricts the value of at least one other, and in general restricts the values of many others [9,12,16]. This property manifests as strong correlations between features, and was the original issue identified by Pearson [16]. The third challenge is that taking sub-compositions of such data often results in completely different interpretations of the correlation structure [12]. Aitchison gives simple examples of this effect where changing the abundance of one feature in a composition results in correlation between the others changing from strongly positive to strongly negative. This effect is problematic, given current guidance by popular 16S tag-sequencing analysis tools to filter reads falling below a certain threshold [19,20] or for removing ribosomal RNA sequences through chemical or computational means when performing RNA-seq.

### Treating compositional datasets as ratios

Aitchison realized that the above constraints could be alleviated by investigating the ratios between proportions [12]. He developed a number of approaches that remove or reduce the above constraints, and with the proper interpretation, allow full use of standard statistical techniques. These transformations have been shown to increase resolution and allow more robust data interpretations in fields as diverse as paleontology [15], environmental sciences [14], metabolomics of wine [13], meta-transcriptomics [8] and 16S rRNA gene tag sequencing [9].

A conceptually simple transformation is the centered log-ratio transformation, or clr. Here the read counts for each feature are divided by the geometric mean of the read counts of each feature in the sample, followed by taking the logarithm. The clr has the advantage that there is a one-to-one transformation of all features, allowing changes of all features to be observed. Moreover, if 2 is used as the base of the logarithm, then differences between features represent fold-changes in relative abundance between features, a measure that is natural for molecular biologists, biochemists and other life scientists. Additional transformations have been developed by Egozcue and collaborators that have more robust properties, but which lack a one-to-one mapping of the sample features [21,22]. We will use the clr transformation throughout the remainder of the paper since this transformation is the most widely used and simplest to interpret.

By way of example, consider the following set of values: *x*=[10,35,50,500]: where the proportional sum is constrained to be 1. We could imagine that we have the counts from an RNA-seq experiment where the last feature is the count of reads mapping to ribosomal RNA. Converting to proportions,

$$\;p_{x}=\frac{10,35,50,500}{595}=\left[0.0168,0.0588,0.0840,0.8403\right]\;,$$

and the difference between elements 1 and 2 is −0.042: a very small difference. If we remove one observation (say the last one), equivalent to what is often done when removing the large number of sequences mapping to rRNA gene sequences from RNA-seq datasets, this changes the dataset to

$$p_{x}=\frac{10,35,50}{95}=\left[0.1052,0.3684,0.5263\right]\;.$$

Now the difference between elements 1 and 2 is much larger, being −0.263, and the investigator could be led to a different conclusion. In contrast we can consider these same elements as compositions and compute the relative difference between elements using the clr. In the case of the complete vector, the corresponding values (using base 2) are clr(*x*)=[−2.44,−0.64,−0.12,3.20] and the difference between elements 1 and 2 is −1.81. If the composition is reduced by removing the last element as before, then the corresponding clr values are [ −1.38,0.43,0.94], and the difference is unchanged at −1.81. The meaning of this result is that element 1 is 2^−1.81^ as abundant as element 2. Thus, the same relative difference between these two elements is maintained regardless of which other element, or combination of elements, is removed.

One additional problem must be acknowledged: that values of zero are problematic because of the logarithmic transformation [12]. Aitchison recommends removing all samples containing a zero value for one of the features when examining geologic samples [12]. This approach is not practical for biologists interested in comparing gene expression between samples or when comparing the differing abundances of bacteria from environmental samples. In these contexts it is quite possible to have features with zero values. These can arise for two reasons. First, it is possible that the feature is truly never represented in the sample. It could be that the organism is incapable of living in that environment or the gene is not expressed. Second, the feature may be present, but below the detection limit imposed by the number of reads that are possible to achieve with the instrument. In the examples that follow, zero values are dealt with in two ways. First, by removing a feature from consideration if the feature has zero counts in all samples. These features are inferred to be so rare that we can assume that sequencing more replicates would always result in zero reads being identified. Second, when one or more values of a composition is greater than zero, then all the values in the composition are retained even if they are zero. We then treat these remaining zeros in a Bayesian context [8,9] and assume that the reason no reads were detected in some features was because of sampling variance. The methodology by which this is done is outlined in the Methods Section, and the full method is implemented in the ALDEx2 R package [23].

## Methods

**Figure 1:**
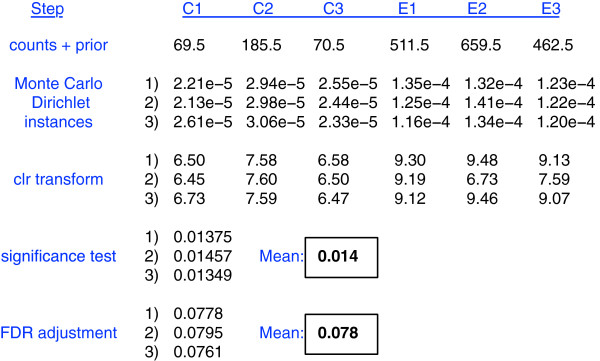
**Outline of the approach for one feature in three control and three experimental samples.** The count values for feature *i*, sample *j* are converted to probabilities by Monte Carlo sampling from the Dirichlet distribution with the addition of a uniform prior. Each count value is now represented by a vector of probabilities 1:*n*, where *n* is the number of Monte Carlo instances sampled: three instances are shown in the example, but 128 are used by default. Each probability in the vector is consistent with the number of counts in feature *i* given the total number of reads observed for sample *j*. Each Monte Carlo Dirichlet instance is center log-ratio transformed giving a vector of transformed values. These values are the base 2 logarithm of the abundance of the feature in each Dirichlet instance in each sample divided by the geometric mean abundance of the Dirichlet instance of the sample. Significance tests for control samples (C1 : C3) vs experimental samples (E1 : E3) are performed on each element in the vector of clr values. Each resulting *P* value is corrected using the Benjamini–Hochberg procedure. The expected values are reported for both the distribution of *P* values and for the distribution of Benjamini–Hochberg corrected values. clr, centered log-ratio; FDR, false discovery rate.

A pictorial summary of the method is outlined in Figure 1. Input data tables for ALDEx2 have *i* rows containing counts of values for each feature, and *j* columns representing samples. Features that contain zero reads in all samples are removed as they are considered uninformative, and by definition are unable to contribute to the pool of differentially abundant features. Similar strategies are standard in RNA-seq analysis where rows summing to less than some value are excluded as they cannot be tested reliably for overdispersion [24]. Similarly, the standard practice in 16S rRNA gene sequencing is to exclude features that are less than an abundance cut-off [19,20], and in practice this usually means excluding singleton reads. The problem is most acute when examining RNA-seq data since structural, ribosomal, transfer and other RNA sequences are physically or computationally depleted prior to analysis. It is crucial to note that when the values in a compositional dataset are manipulated in this way the data transformations proposed by Aitchison and used here are required to prevent spurious conclusions because of sub-compositional incoherence in compositional data [12]. The total number of reads for all features in a sample $N_{j}=\underset{i}{\sum }n_{i,j}$ is not a predictable outcome of the experimental design because it is dependent on the instrument capacity and the number of samples that are multiplexed in the run. The actual number of counts for a given feature are therefore not of interest and are generally scaled [2]. Note that along with the total counts per sample, the total number of counts does provide information about the precision of per feature count estimation [8,9].

Normalization across samples can be achieved using maximum likelihood to give a single estimate of the proportion of reads per feature, $p_{i,j}=n_{i,j}/\underset{i}{\sum }n_{i,j}$. However, this simple normalization has several flaws, mainly that both high and low count features may not be estimated correctly [2,25]. The number of reads per feature can be modeled as being sampled from a multinomial Poisson process, and the approach that performs best is to model the read counts as being derived from a Dirichlet process [8,9,26,27].

Figure 1 shows a worked example for a single feature *i* for three control (C) and three experimental (E) samples. First, sequences that map to each feature are enumerated and the table of read counts for each feature in each sample is converted to a distribution of posterior probabilities through Monte Carlo sampling from the Dirichlet distribution for each sample:

$$p\;\left[n1,n2,\ldots \right]|\sum N=\mathrm{Dir}\;\left(\left[n1,n2,\ldots \right]+\frac{1}{2}\right)\;.$$

An uninformative prior of 1/2 is used to model the frequency of features with zero counts [28,29]. This prior maximizes the information in the data while minimizing the effect of the prior on the posterior in the case where the relative frequencies of each feature are of equal importance [29]. Usually, 128 Dirichlet Monte Carlo (DMC) instances are sufficient since we are concerned only with summarizing central tendencies, not tail-related events. Each point value is now represented by a vector of posterior probabilities, *p*_*i*,*j*_[1,2,…]. The distributions are narrow if the feature and the sample contain a large number of counts and wide if either the feature or the sample has a small number of counts. Each Monte Carlo realization of *p*_*i*,*j*_ is transformed by the clr transformation:

$$c_{i,j}=\log_{2}\;(p_{i,j})-\text{mean}\log_{2}\;(p_{j}).$$

After this transformation the value for each feature is now relative to the geometric mean abundance of all values in the sample. We will refer to clr-transformed values as relative abundance values, and to untransformed values as proportional values throughout. Each realization of the *c*_*i*,*j*_ value between conditions is subjected to both a Welch’s *t*-test and a Wilcoxon rank test giving two vectors of *P* values, and each *P* value instance is then corrected for multiple hypothesis testing using the false discovery rate (fdr) approach of Benjamini and Hochberg [30]. The expected value of the *P* and fdr statistics are then reported for both statistical tests.

ALDEx2 also returns the within- and between-condition measures and the effect size that was used by the original version of ALDEx [8]. However, these statistics are calculated in a much more efficient way allowing near arbitrarily sized experimental designs. The major differences between ALDEx2 and the original version are outlined in Table 1.

**Table 1:** Comparison of ALDEx and ALDEx2

**Property**	**ALDEx**	**ALDEx2**
Significance tests	Effect size and ad hoc	Welch’s *t* or Wilcoxon
Multiple test	No	Benjamini–Hochberg
correction		
Minimum dataset	Two samples per	Three samples per
	group	group
Maximum dataset	3×3×5, 000	>300×300×20, 000
	2×2×20, 000	

Dataset: samples in A × samples in B × number of features.

The default parameters of ALDEx 2.0.6, DESeq v1.14.0 [31], DESeq2 v1.2.8 [31], SAMseq v2.0 [32] and baySeq v1.16.0 [33] versions were used in R version v3.0.2 [34].

## Results and discussion

We will use example datasets that are sufficiently different to show that the concepts are generalizable to many types of high-throughput sequencing studies including selective growth experiments, RNA-seq, ChIP seq, 16S rRNA gene tag sequencing and others.

### Examining selective growth experiments

Selective growth experiments (selexes) are often used to identify sequence variants for genes that confer a growth phenotype upon the cell line containing them. In this type of experiment the investigator is interested in identifying the fold enrichment of variants that exhibit some activity.

The first dataset is a single-round enrichment experiment testing the activity of a library of LAGLIDADG homing endonuclease sequence variants [35]. This dataset is available within the ALDEx2 package. In this experiment two codons in the gene were randomized completely and two codons were free to encode only two acid amino acids. The total library was thus 1,600 possible quadruple amino acid variants. An active enzyme results in cleavage of the gene for a bacteriostatic DNA gyrase toxin. DNA sequencing was used to collect data on the growth characteristics of cells containing individual variants in the library under both selective and non-selective (i.e., no toxin) conditions. Seven replicate experiments for activity were conducted. With this experimental design it is expected that the relative abundances of each variant after growth in the non-selective condition would reflect the input abundances and vary only by sampling differences. Crucially, because the toxin is bacteriostatic, the relative abundances of each variant under the selective conditions should also reflect the input abundances since all variants should survive but no variant should be able to grow. Thus, their relative abundance would be unchanged. In contrast, variants that allow escape from the selection would become relatively more abundant, but no variant becomes less abundant.

This dataset is useful because the directionality of change is fixed, and because the activity of some of the variants has been verified *in vitro* giving an objective measure of truth that is often lacking in sequence survey experiments such as 16S rRNA gene sequencing or RNA-seq. We use this dataset to illustrate the advantages of compositional data analysis using the clr transformation, the effect of modeling low count abundances with Monte Carlo replicates of the Dirichlet distribution and the effect of sample size on power.

#### Analyzing selective growth data as counts

Among the most developed tools to examine high-throughput sequencing experiments are those designed to examine RNA-seq experiments [1], and these tools are often advocated for use in other experimental designs that generate tables of counts. These tools assume the data are counts of features for each sample and scale the counts to account for sequencing depth. The variance of each feature is calculated from a negative binomial model. We started with the hypothesis that existing tools used to examine RNA-seq experiments would be appropriate to identify those variants that exhibit differential growth. We tested this hypothesis using DESeq, DESeq2 [1], SAMseq [32] and baySeq [33] to identify differentially abundant variants. Table 2 shows that an examination of the data at a fdr of 5% indicated that the majority, or in some cases all, of the amino acid variants exhibited a significantly different frequency pre- and post-selection with each of these tools. All four tools identified a small number of features as becoming more abundant, and a large number of features as becoming less abundant. This makes sense in the context of counts, but not in the context of relative abundance because the vast majority of variants do not change their relative abundance under the selective conditions. Therefore, the differential growth exhibited by the variants under the selective and non-selective growth conditions clearly does not fit the underlying assumptions of any of these tools.

**Table 2:** ***In vitro* selection experiment analysis of commonly used differential expression tools**

**Tool**	**Number up**	**Number down**
BaySeq	8	1,592
SAMseq	7	708
DESeq	21	1,396
DESeq2	32	1,400
ALDEx2	69	0

#### Analyzing selective growth data as compositions

The analysis that follows uses the clr transformation, described in the Methods, which converts the data from absolute differences to relative differences. Data transformed by the clr are centered on the geometric mean, with negative values being less abundant than the mean in that sample and positive values being more abundant than the mean. The difference between two values is the fold-change between them if the transformation is done using base 2 logarithms. One important property of the clr transformation is that the transformed values are inherently normalized for the sequencing effort [8].

Several groups have recently shown that the sampling error inherent in high-throughput sequencing protocols can be modeled appropriately by Monte Carlo sampling from a Dirichlet distribution [8,9,26,27]. Each instance sampled from a Dirichlet distribution is equivalent to assuming that read proportions for each feature are derived from a Poisson distribution with the additional constraint that the sum of the proportions of all compositions must sum to 1 after sampling [28]. This approach gives a more accurate assessment of the expected value of the proportion for a given composition, and a correspondingly more accurate estimate of the expected value of the associated statistical values [8,9]. Monte Carlo instances can be drawn from the Dirichlet distribution and each Dirichlet instance is an estimate of the per feature proportion that would be observed if the same library had been sequenced again [8]. In effect, each Dirichlet instance is an equally valid outcome of the sequencing run based upon the total number of reads observed and their per feature distribution. One consistent problem with conducting experiments where there are hundreds or thousands of features is that the number of hypothesis tests conducted are greater than the degrees of freedom allowed by the number of replicates. This problem, in addition to the large uncertainty in accurately measuring the frequency of low-abundance features, means that it is very easy to underestimate the true underlying variation in the data, and to overestimate statistical significance [8,9].

**Figure 2:**
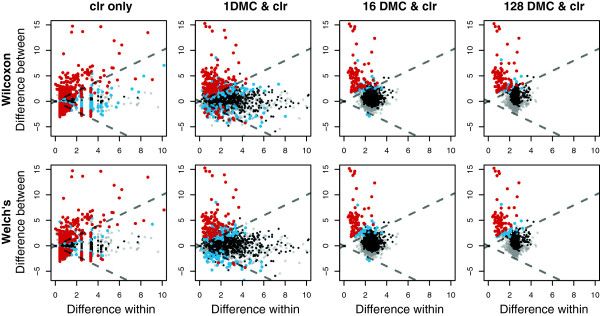
**Effect of DMC sampling on the selex dataset.** The first column shows the results when the data is clr transformed without DMC sampling, the next three show the effect of 1, 16 and 128 DMC samples followed by the clr transformation. Features that pass a threshold *P*<0.05 are shown in cyan and those where the fdr statistic is <0.05, are shown in red. Features where the median clr value is below the geometric mean are highlighted in black if they are not significant. Those where the median clr value is greater than the geometric mean are shown in gray. clr, centered log-ratio; DMC, Dirichlet Monte Carlo.

Figure 2 shows plots of the between-condition variation versus the within-condition variation (MW plots) [8] for clr-transformed data alone, or with the expected valued derived from differing numbers of DMC instances. Note that the MW plots show that there are many more ‘significant’ features (red) when the clr transformation is used in the absence of DMC instances (clr only) or when there is only a single DMC instance than when the expected values of the test statistics are determined from 16 or 128 instances. This occurs with both statistical tests.

The low-abundance features, shown in black, have a very wide spread in their within-condition differences in the clr only example and when only a single DMC instance is used. The effect of determining the expected values through DMC sampling is evident in the two right columns, where it can be seen that the within-condition differences for low-abundance features converge when the number of instances increases. This indicates that small numbers of samples are insufficient to reflect adequately the true sampling variation [8,9], especially for the rare features that exhibit high relative variation. It is apparent that determining the expected value of the test statistics from multiple Monte Carlo realizations has a profound effect on the estimation of variance in those features with low abundance values as these features (shown in black) are largely displaced further to the right, being on average almost twice as variable in this procedure than when only the clr transformation is performed.

Table 3 enumerates the number of low-abundance features identified as significant as a fraction of the total number of significant features and the number of Monte Carlo samples from the Dirichlet distribution (DMC). Note that low-abundance features compose a large fraction of all significant features when the clr only is used and when only a single DMC is used. Both the total number of significant features and the contribution of low-abundance features drops rapidly when DMC replicates are used to estimate the sampling variation. This is similar to the observation of Friedman and Alm [9], who demonstrated that examining expected values of test statistics derived from DMC sampling significantly reduced the number of false positive correlated features in 16S rRNA gene tag sequencing experiments.

**Table 3:** Effect of Dirichlet Monte Carlo instances on significance of low-abundance features

**DMC**	**Wilcoxon**^**a**^	**Welch’s**^**a**^
0	339/478	349/546
1	41/164	27/111
16	1/84	0/68
128	0/85	0/69

DMC, Dirichlet Monte Carlo replicate number. ^a^Number of low-abundance significant features/total.

One shortcoming of the clr transformation is that it may not transform the data such that it is normally distributed. In this case it may be more appropriate to use a non-parametric significance test to determine the difference between conditions. The top and bottom rows in Figure 2 show the results of Wilcoxon tests or of Welch’s *t*-tests on the clr-transformed values. We found that there was very good agreement between features identified by both statistical tests when using all seven replicates since all 69 features identified by the Welch’s *t*-test were in the 85 features identified by the Wilcoxon test in the 128 DMC analysis using all seven replicates at a fdr <0.05. However, when only three of the seven replicates were included, no features were identified as significant by the Wilcoxon test while 16 features were still identified as significant by Welch’s *t*-test. We therefore recommend Welch’s *t*-test as its power is not as sensitive to sample size as the Wilcoxon test, although both test statistics are reported by ALDEx2, and the user should examine the results of both tests.

#### Examining the results of count vs composition analysis

The last line in Table 2 shows that all of the significant features identified by the ALDEx2 approach are identified as becoming relatively more abundant, and, in contrast to the other tools, no features are identified as becoming relatively less abundant. That DESeq, DESeq2, SAMseq and baySeq identify features that become less abundant makes sense if the data are counts, but do not make sense if the data are compositional. Recall that the design of the experiment was to identify those sequence variants that exhibited differential effects in a bacteriostatic growth assay. Thus if a variant was non-functional the abundance of the variant would be unchanged in the selected condition; i.e., cells containing a non-functioning variant would not grow and so these cells would remain at the input concentration until sampled. Conversely, if a variant was functional, cells containing it would grow and become much more abundant than average under the selective conditions. Note that cells containing both functional and non-functional variants would grow at the same rate and so the expectation is that variants would be found at approximately the same abundance under non-selective conditions. By way of illustration consider two variants from the dataset. The first variant K:D:I:E is non-functional, the counts for non-selected cells are [ 149,89,165,68,135,128,199] and for selected cells are [ 0,0,1,0,1,0,0]. Here the counts are very different, leading to the conclusion that this feature is less abundant in the selected than the unselected dataset, and this is reported as such by all tools (fdr for DESeq and baySeq are 3.8×10^−7^ and 1.8×10^−4^, respectively). However, *relative to all other variants* the counts for this variant are almost equal to the geometric mean abundance in both the selected and non-selected conditions: ALDEx2 reports an fdr of approximately 0.8 indicating no difference relative to the mean abundance under each condition. The second variant, S:E:G:D, is weakly functional and the counts for non-selected cells are [ 755,554,669,797,862,650,2170] and for selected cells are [ 4710,995,906,1716,784,804,641]. This variant is identified as non-signficant by DESeq (fdr = 0.73), but is considered significant by ALDEx2 (fdr <1×10^−3^). The difference is presumably because the counts are similar in the selected and non-selected conditions (DESeq log2(fold-change)=2^2.9^), but the relative abundances are very different (ALDEx2 diff.btw.q 50=2^6.2^).

We conclude that these tools, and by extension the many similar tools that use count-based methods to estimate variation [2,36], are inappropriate as general-purpose tools to examine differential growth experiments of this type. In contrast the clr transformation in combination with Dirichlet sampling indicated that only a minority of the variant combinations were under strong positive selection, a result that was in agreement with the biochemical characterization of the variants [35].

### Analyzing RNA-seq data as compositions

The second dataset was generated by Bottomly *et al*. [37] and contains 10 biological replicates of RNA isolated from the brain striatum from one mouse strain (C57BL6J) and 11 biological replicates of another strain (DBA/2J). The dataset contains an average of 22 million reads mapped for each sample. It was accessed from the ReCount dataset [38] in January 2014. The final output from ALDEx2 is available as Additional file 1.

RNA-seq attempts to address the question: ‘What are the differences between gene expression in the samples in two or more conditions?’ It is reasonable to examine gene expression values as fold-change (relative differences) because the law of mass action that governs biochemical reactions depends on the ratios of reactants. For this reason, existing RNA-seq analysis tools report changes in gene expression as fold differences, despite computing *P* values as differences in scaled counts. What is not often appreciated is that gene expression is itself a limited resource in the cell, meaning that the transcript abundance should also be modeled as proportional [39]. Furthermore, it is standard practice in the field to sequence only a subset of the total RNA in a sample: typically only the mRNA or some types of non-coding RNA are sequenced and rRNA and tRNA are excluded. This approach dramatically alters the sub-compositional structure of the data, potentially leading to non-robust inferences as discussed in the introduction.

We examined the Bottomly *et al*. RNA-seq dataset [37] using the parametric tools DESeq and baySeq and compared these results to those obtained by ALDEx2, which treats the dataset as being compositional rather than count based. Both DESeq and baySeq were recently shown to be among the most conservative when examining this dataset [36], and so can be considered to have assumptions that have been iteratively altered to fit to the underlying data better than the majority of tools used for this purpose. The interested reader should see Soneson and Delorenzi [36] for a detailed examination of a large number of available tools using this dataset. These tools, along with a majority of RNA-seq analysis tools, use the negative binomial distribution to estimate the variance of gene expression abundance as a function of the expression. This method was used originally because high sequencing costs constrained the number of replicate samples. Indeed, many published analyses contain three or fewer replicates. The negative binomial approach allowed the estimation of the variance within a condition as a function of the mean expression [31]. This variance, estimated from the idealized negative binomial, is then used to assign *P* values and to calculate corresponding fdr statistics. Thus, this approach allowed the estimation of statistical significance when the number of samples was small.

**Figure 3:**
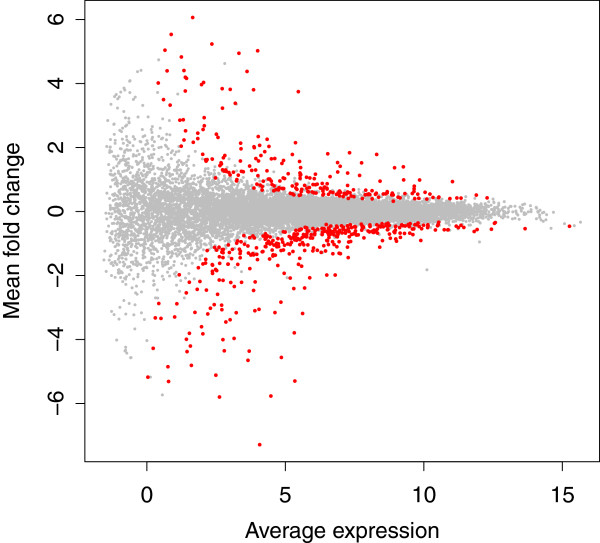
**MA plot for DESeq.** The base 2 logarithm of average expression across all samples for a feature is plotted vs the base 2 logarithm of fold-change. Points that are significantly different with a fdr less than 0.05 are in red, all others are in gray.

Differential expression in RNA-seq datasets is often visualized globally by Bland-Altman plots [40], which show the mean difference (M) vs average expression (A) (MA plots). An example MA plot derived from the DESeq package [31] for the large RNA-seq dataset presented by Bottomly *et al*. [37] is shown in Figure 3. In this figure the points corresponding to the genes identified as differentially expressed are colored red. Note the relationship between fold-change and mean expression: genes with a small fold-change require high expression, and genes with a low expression value require a high fold-change to be identified as differentially expressed. Note also that for a given fold-change and expression value, the majority of genes with a greater fold-change and expression value are identified as differentially expressed. This MA plot is typical of well-behaved datasets and such an idealized MA plot suggests that the analysis by this tool is valid. In this dataset 604 genes were identified as differentially abundant between conditions by DESeq using an fdr cut-off of 0.05.

**Figure 4:**
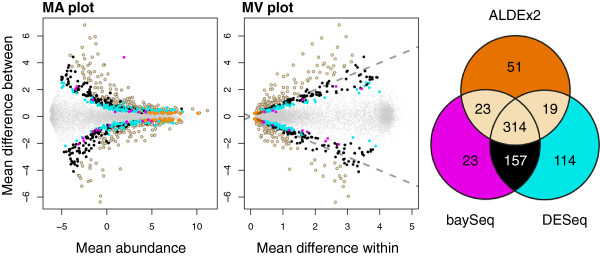
**Differential features in common between ALDEx2, DESeq and baySeq.** Genes are colored in light yellow if ALDEx and at least one of the other two tools identified them as significantly different with an fdr <0.05, black if they were identified by both baySeq and DESeq, magenta if only by baySeq, cyan if only by DESeq, and orange if only by ALDEx2. Small gray dots are non-differential genes. The Venn diagram illustrates the number of differentially abundant genes identified by each method. MA, mean difference between conditions vs average expression; MW, mean difference between conditions vs maximum within-condition variance.

The MA plot in Figure 3 was plotted using values output by DESeq, which assumes that the underlying data was count data and not compositional data [1]. We examined the similarity between the DESeq and ALDEx2 underlying values in two ways. First, by comparing the DESeq corrected mean count values and the ALDEx2 median abundance values, and by comparing the DESeq and ALDEx2 fold-change values. There was an almost perfect linear relationship for both these examples, with a Spearman correlation of >0.99 in both cases. Second, we replotted the values from DESeq onto clr space, and show these plots in the two left panels of Figure 4. In this figure features identified as significant by DESeq, baySeq and ALDEx2 individually and in common are highlighted in different colors: features identified as significant by DESeq are given by the filled light yellow circles along with the filled black and cyan circles; features identified as significant by baySeq are given by the filled light yellow circles and the filled black and magenta circles; and features identified by ALDEx2 (and at least one of the other two tools) are given by the filled light yellow circles and the orange filled circles. It is obvious from the left panel that the majority of genes identified by DESeq and baySeq as differentially expressed are consistent with the remapped sample space.

The MW plot panel in Figure 4 shows how the variation in the dataset is distributed within and between conditions. In this plot the position where there is equal variation within and between conditions is shown by the dashed lines. The coloring is the same as for the left panel. The MW plot shows that a large fraction of the significant genes identified by both DESeq and baySeq (in black), by DESeq only (in cyan) or baySeq only (in magenta) exhibit as much or more within-condition variation than between-condition differences. This is not a statistically desirable result. The genes identified as differential by DESeq or baySeq individually tend to be even more variable within a condition than those identified by both tools. In contrast, the significant features identified by ALDEx2 (in black and orange) always have a larger between-condition difference than within-condition difference. Those genes that are uniquely identified by ALDEx2 (in orange) are almost exclusively genes with very high expression and relatively small differences between conditions.

The number of significant features identified by DESeq, baySeq and ALDEx2 is presented in a Venn diagram in the third panel of Figure 4. It is apparent that all three methods identified approximately the same number of genes as being differentially abundant (ALDEx2 407, baySeq 517, DESeq 604) when controlling for an fdr of 0.05, and between 52% and 77% of the genes identified by one method were identified by all three. In summary, it can be seen that the expected value of the *t*-test for DMC-sampled and clr-transformed data ensures that the genes identified as differentially expressed exhibit a greater gene expression difference between mouse strains than the gene expression variation within either mouse strain. This example shows that the compositional analysis approach works well for RNA-seq datasets.

### Analyzing 16S rRNA gene tag sequencing data as compositions

The third dataset is a 16S rRNA gene sequencing dataset obtained from the Human Microbiome web repository on 10 January 2014 [41]. This dataset is a highly curated and annotated set of 16S sequence counts collected from a large number of people and a large number of body sites. We chose to examine the microbiota composition differences between the tongue dorsum and buccal mucosa because these sites had been shown to have somewhat different microbial compositions both by a comprehensive analysis of the same 16S rRNA gene data and by an independent shotgun metagenomics analysis [42]. Since both approaches identified substantially similar taxa as differentially abundant, we were interested to determine if the relatively simple procedure implemented here could recapitulate the analyses done previously. The final output from ALDEx2 is available as Additional file 2.

**Figure 5:**
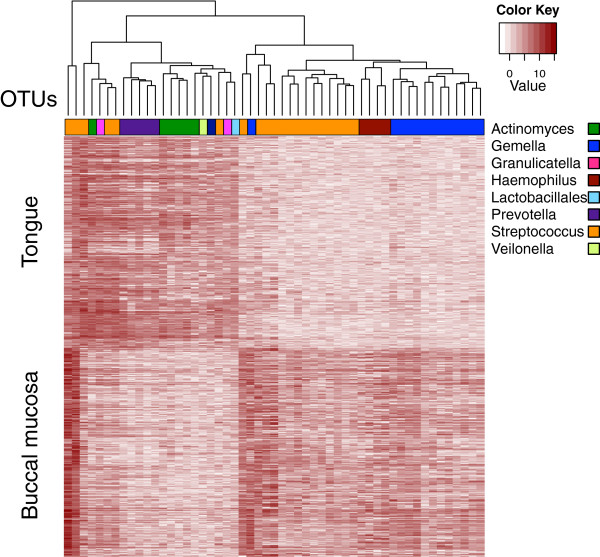
**OTUs with different relative abundances between tongue dorsum and buccal mucosa.** Each OTU is colored by membership in the taxonomic level indicated. OTU abundance values are median relative abundance values derived from ALDEx2. OTU, operational taxonomic unit.

The Human Microbiome Project dataset contained 23,393 OTUs containing one count or more for any of the 316 samples from the tongue dorsum and 312 samples from the buccal mucosa. This dataset was analyzed with 16 DMC instances rather than 128 because of time and memory constraints, and because the analysis above suggested that 16 DMC samples would provide sufficient selectivity on low-count OTUs. The analysis was completed over all 23,393 OTUs in the dataset in approximately 76 minutes on a computer with 16 Gb RAM and an i7 processor. We observed 755 differentially abundant OTUs that had a Benjamini–Hochberg fdr <0.05, and collected the 53 OTUs that passed that fdr cut-off with absolute effect size >0.6. These 53 OTUs are displayed on the heat map in Figure 5.

The results show that OTUs assigned to the genera *Actinomyces*, *Prevotella* and *Veillonella* have an increased relative abundance in samples from the tongue dorsum, while OTUs assigned to *Haemophilus* and *Gemella* have an increased relative abundance in samples from the buccal mucosa. Several distinct OTUs assigned to *Streptococcus* had different relative abundances in both body sites, although, in general OTUs assigned to this genus were over-represented in the buccal mucosa. Together, these results are congruent with those of Segata *et al.*, who observed that these genera differentiate these body sites [42]. We conclude that the statistical procedure is also applicable to identify taxa that are differentially abundant between two samples in 16S rRNA gene tag sequencing experiments.

## Conclusions

High-throughput sequencing is increasingly used to identify differences between datasets composed of DNA or RNA sequences. Here we show that data transformations appropriate for compositional data [12] can be used with standard statistical inference tools, such as Welch’s *t*-test or the non-parametric Wilcoxon test, to identify features that are differentially abundant between conditions in datasets derived from selexes, RNA-seq or 16S rRNA gene segment sequencing. Historically, each of these experimental designs has used a distinct statistical model to determine significance when examining difference between conditions, despite these experiments generating similar types of data comprising large numbers of reads that are binned into one or more categories.

The diversity of methods suggests a situation where the assumptions and parameters have been tuned to give biologically meaningful results with the unintended consequence of making these methods unexpectedly fragile. For example, RNA-seq analysis tools are very sensitive to outliers in the datasets [8,32]. Recognizing this, Li and Tibshirani developed SAMseq, which essentially performs a consistency check in a manner similar to ALDEx2. However, in their approach sampling is performed using a Poisson model that does not enforce a constant sum constraint, and they use the Wilcoxon rank test, which requires a large number of samples for statistical power. Furthermore, SAMseq treats the data as counts rather than compositions. Nevertheless, this method was shown to be superior to existing RNA-seq analysis tools based on Poisson or negative binomial models [32]. While this approach is superior in some ways to existing RNA-seq analysis tools on specific datasets, this tool set does not translate well to other experimental designs as shown in Table 2. In addition, RNA-seq analysis tools differ in how the values in the data are scaled between samples, and there is debate in the literature as to which scaling method is generally superior [2]. Finally, modeling has failed to reveal the ‘best’ tool because the tool that performs the best varies widely when the parameters of the model are altered [36].

For 16S rRNA gene sequencing, although the tools used to determine differential abundance between conditions are still evolving rapidly, no tool treats the data as compositions and ensures sub-compositional coherence. For example, one widely used method [5] is similar to SAMseq in that it models the reads as coming from an underlying Poisson model to estimate technical variance and identifies as differential those features that show significance that is not sensitive to the estimated variance. However, this method treats the values as proportions and not as compositions. Another commonly used transformation in 16S rRNA gene sequencing is the Hellinger transformation [43] implemented in the VEGAN R package [44]. This transformation is also count based and is not sub-compositionally coherent.

Demonstration of the superiority of analysis methods is limited by two main factors: the first is that the abundance of a feature in a sample is a continuous variable, and the second is that there is often no objective standard to determine what is differentially abundant. Without *a priori* information on what constitutes a biologically meaningful difference, there is no clearly demarcated line-in-the-sand that can be drawn between differential and non-differential abundance in any experimental design. Moreover, the relatively high cost of sequencing has led to study designs that often emphasize per sample sequencing depth over biological replication [45], despite advice to the contrary [46,47]. This has led to the development of tools that attempt to estimate the biological variation from limited numbers of replicates.

Standard statistical practice indicates that the analyses should be limited to determining those features with abundance differences between conditions that are reliably larger than the variation within either condition. We and others have developed non-parametric tools to identify those features fulfilling those criteria [8,32].

The approach used in this paper models the reads per feature as proportions, acknowledging that the total sum of reads for a sample is not itself important. The precision of estimation of the proportional values is determined by taking Monte Carlo instances sampled from the Dirichlet distribution, which takes into account the number of reads per feature and the total sequencing depth. This approach generates narrow distributions when the read count is high per feature and per sample. The proportional data are transformed using the clr transformation, allowing standard statistical tools to be applied to each instance. Summary statistics are then reported as the expected value of the distributions.

We show that this approach is generalizable to three completely different experimental designs: a selex, an RNA-seq type experiment and a 16S rRNA gene amplicon-sequencing experiment. For the selex experiment, the ALDEx2 approach identified known active enzyme variants and weeded out inactive variants [35]. ALDEx2 identified a set of operational taxonomic units differential between two closely positioned body sites consistent with the results of two independent methods in the literature [42]. For RNA-seq, the ALDEx2 approach identified essentially all genes found by both DESeq and baySeq where the inter-condition difference was larger than the intra-condition variance. We believe that ALDEx2 exhibited greater specificity and equivalent sensitivity as these widely used tools.

## Abbreviations

ChIP-seq: Chromatin immunoprecipitation sequencing; clr: centered log-ratio; DMC: Monte Carlo instance of the Dirichlet distribution; fdr: false discovery rate; MA: Mean difference between conditions vs average expression; MW: Mean difference between conditions vs maximum within-condition variance; OTU: Operational taxonomic unit; PCR: Polymerase chain reaction; PPM: Parts per million; RAM: Random access memory; RNA-seq: RNA sequencing; rRNA: Ribosomal RNA; selex: selective growth experiment.

## Competing interests

The authors declare that they have no competing interests.

## Authors’ contributions

AF conceived and designed the study, analyzed the data, contributed code and gave final approval for the manuscript. GG conceived and designed the study, analyzed the data, contributed code, wrote the manuscript and gave final approval for the manuscript. JM analyzed the data, contributed code to ALDEx2, revised the manuscript and gave final approval for the manuscript. TM designed the study, generated and validated the datasets and gave final approval for the manuscript. DE designed the study, generated and validated the datasets, revised the manuscript and gave final approval for the manuscript. JR designed the study, analyzed the data, revised the manuscript and gave final approval for the manuscript. All authors read and approved the final manuscript.

## Supplementary Material

Additional file 1 **Excel file containing ALDEx2 output for the Bottomly RNA-seq dataset.** Column 1 contains gene features from the mouse genome. Column 2 contains the relative abundance of each feature averaged across all samples. Columns 2 and 3 contain the average relative abundance of DBA/2J and C57BL6J gene expression. Columns 4 and 5 contain the median between- and within-condition differences. Column 6 contains the median effect size. Columns 7 and 8 contain the expected values of *P* and the associated Benjamini–Hochberg corrected fdr values for Welch’s *t*-tests and the final two columns contain the expected values for Wilcoxon tests. Click here for file

Additional file 2 **Excel file containing ALDEx2 output for the buccal vs tongue dorsum comparison.** Column 1 contains HMP OTU identifier labels. Column 2 contains the relative abundance of each feature averaged across all samples. Columns 2 and 3 contain the average relative abundance of buccal and tongue OTU sequences. Columns 4 and 5 contain the median between- and within-condition differences. Column 6 contains the median effect size. Columns 7 and 8 contain the expected values of *P* and the associated Benjamini–Hochberg corrected fdr values for Welch’s *t*-tests and the final two columns contain the expected values for Wilcoxon tests. Click here for file

## References

[B1] Anders S McCarthy DJ Chen Y Okoniewski M Smyth GK Huber W Robinson MD Count-based 631 differential expression analysis of RNA sequencing data using R and Bioconductor Nat Protoc 2013 8 9 1765 86 10.1038/nprot.2013.099 23975260

[B2] Dillies M-A Rau A Aubert J Hennequet-Antier C Jeanmougin M Servant N Keime C Marot G Castel D Estelle J Guernec G Jagla B Jouneau L Laloë D Le Gall C Schaëffer B Le Crom S Guedj M Jaffrëzic F on behalf of the French StatOmique Consortium A comprehensive evaluation of normalizationmethods for Illumina high-throughput RNA sequencing data analysis Brief Bioinform 2013 14 6 671 83 10.1093/bib/bbs046 22988256

[B3] Schloss PD Westcott SL Ryabin T Hall JR Hartmann M Hollister EB Lesniewski RA Oakley BB Parks DH Robinson CJ Sahl JW Stres B Thallinger GG Van Horn DJ Weber CF Introducing mothur: open-source, platform-independent, community-supported software for describing and comparing microbial communities Appl Environ Microbiol 2009 75 23 7537 41 10.1128/AEM.01541-09 19801464 PMC2786419

[B4] Caporaso JG Kuczynski J Stombaugh J Bittinger K Bushman FD Costello EK Fierer N Peña AG Goodrich JK Gordon JI Huttley GA Kelley ST Knights D Koenig JE Ley RE Lozupone CA McDonald D Muegge BD Pirrung M Reeder J Sevinsky JR Turnbaugh PJ Walters WA Widmann J Yatsunenko T Zaneveld J Knight R Qiime allows analysis of high-throughput community sequencing data Nat Methods 2010 7 5 335 6 10.1038/nmeth.f.303 20383131 PMC3156573

[B5] Faust K Sathirapongsasuti JF Izard J Segata N Gevers D Raes J Huttenhower C Microbial co-occurrence relationships in the human microbiome PLoS Comput Biol 2012 8 7 1002606 10.1371/journal.pcbi.1002606 PMC3395616 22807668

[B6] Smith CJ Osborn AM Advantages and limitations of quantitative PCR (Q-PCR)-based approaches in microbial ecology FEMS Microbiol Ecol 2009 67 1 6 20 10.1111/j.1574-6941.2008.00629.x 19120456

[B7] Zuo C Keles S A statistical framework for power calculations in ChIP-seq experiments Bioinformatics 2013 30 6 753 60 23665773 10.1093/bioinformatics/btt200 PMC3957067

[B8] Fernandes AD Macklaim JM Linn TG Reid G Gloor GB ANOVA-like differential expression (ALDEx) analysis for mixed population RNA-seq PLoS ONE 2013 8 7 67019 10.1371/journal.pone.0067019 PMC3699591 23843979

[B9] Friedman J Alm EJ Inferring correlation networks from genomic survey data PLoS Comput Biol 2012 8 9 1002687 10.1371/journal.pcbi.1002687 PMC3447976 23028285

[B10] Kuczynski J Lauber CL Walters WA Parfrey LW Clemente JC Gevers D Knight R Experimental and analytical tools for studying the human microbiome Nat Rev Genet 2012 13 1 47 58 22179717 10.1038/nrg3129 PMC5119550

[B11] Lovell D Müller W Taylor J Zwart A Helliwell C Pawlowsky-Glahn V Buccianti A Pawlowsky-Glahn V, Buccianti A Proportions, percentages, ppm: do the molecular biosciences treat compositional data right? Compositional Data Anal: Theory Appl 2011 Chichester: John Wiley & Sons 193 207

[B12] Aitchison J The Statistical Analysis of Compositional Data 1986 London: Chapman & Hall

[B13] Hron K Jelínková M Filzmoser P Kreuziger R Barták P Bednář P Statistical analysis of wines using a robust compositional biplot Talanta 2012 90 46 50 22340114 10.1016/j.talanta.2011.12.060

[B14] Filzmoser P Hron K Reimann C Univariate statistical analysis of environmental (compositional) data: problems and possibilities Sci Total Environ 2009 407 23 6100 8 10.1016/j.scitotenv.2009.08.008 19740525

[B15] Kucera M Malmgren BA Logratio transformation of compositional data: a resolution of the constant sum constraint Mar Micropaleontology 1998 34 1 117 20

[B16] Pearson K Mathematical contributions to the theory of evolution – on a form of spurious correlation which may arise when indices are used in the measurement of organs Proc R Soc Lond 1896 60 489 98 10.1098/rspl.1896.0076

[B17] van den Boogaart KG Tolosana-Delgado R ‘compositions’: a unified R package to analyze compositional data Comput Geosci 2008 34 4 320 38 10.1016/j.cageo.2006.11.017

[B18] Efron B Nonparametric estimates of standard error: the jackknife, the bootstrap and other methods Biometrika 1981 68 3 589 10.1093/biomet/68.3.589

[B19] Gloor GB Hummelen R Macklaim JM Dickson RJ Fernandes AD MacPhee R Reid G Microbiome profiling by Illumina sequencing of combinatorial sequence-tagged PCR products PLoS One 2010 5 10 15406 10.1371/journal.pone.0015406 PMC2964327 21048977

[B20] Caporaso JG Lauber CL Walters WA Berg-Lyons D Lozupone CA Turnbaugh PJ Fierer N Knight R Global patterns of 16s rRNA diversity at a depth of millions of sequences per sample Proc Natl Acad Sci USA 2011 108 (Suppl 1 4516 22 20534432 10.1073/pnas.1000080107 PMC3063599

[B21] Egozcue J Pawlowsky-Glahn V Groups of parts and their balances in compositional data analysis Math Geol 2005 37 7 795 828 10.1007/s11004-005-7381-9

[B22] Egozcue JJ Pawlowsky-Glahn V Mateu-Figueras G Barcelõ-Vidal C Isometric logratio transformations for compositional data analysis Math Geol 2003 35 3 279 300 10.1023/A:1023818214614

[B23] ALDEx2 R package [https://github.com/ggloor/ALDEx2]

[B24] Auer PL Doerge RW A two-stage Poisson model for testing RNA-seq data Stat Appl Genet Mol Biol 2011 10 1 1 26

[B25] Newey WK McFadden D Engle R, McFadden D Large sample estimation and hypothesis testing Handbook of Econometrics. Volume 4 1994 Amsterdam: Elsevier Science 2111 245

[B26] Holmes I Harris K Quince C Dirichlet multinomial mixtures: generative models for microbial metagenomics PLoS One 2012 7 2 30126 10.1371/journal.pone.0030126 PMC3272020 22319561

[B27] La Rosa PS Brooks JP Deych E Boone EL Edwards DJ Wang Q Sodergren E Weinstock G Shannon WD Hypothesis testing and power calculations for taxonomic-based human microbiome data PLoS One 2012 7 12 52078 10.1371/journal.pone.0052078 PMC3527355 23284876

[B28] Frigyik BA Kapila A Gupta MR Introduction to the Dirichlet distribution and related processes Technical Report UWEETR-2010-0006, Department of Electrical Engineering, University of Washington December 2010 [https://www.ee.washington.edu/techsite/papers/refer/UWEETR-2010-0006.html]

[B29] Berger JO Bernardo JM Ordered group reference priors with application to the multinomial problem Biometrika 1992 79 1 25 10.1093/biomet/79.1.25

[B30] Benjamini Y Hochberg Y Controlling the false discovery rate: a practical and powerful approach to multiple testing J R Stat Soc Series B (Methodol) 1995 57 1 289 300

[B31] Anders S Huber W Differential expression analysis for sequence count data Genome Biol 2010 11 10 106 10.1186/gb-2010-11-10-r106 20979621 PMC3218662

[B32] Li J Tibshirani R Finding consistent patterns: a nonparametric approach for identifying differential expression in RNA-seq data Stat Methods Med Res 2013 22 5 519 36 10.1177/0962280211428386 22127579 PMC4605138

[B33] Hardcastle TJ Kelly KA Empirical Bayesian analysis of paired high-throughput sequencing data with a beta-binomial distribution BMC Bioinformatics 2013 14 1 135 10.1186/1471-2105-14-135 23617841 PMC3658937

[B34] R Development Core Team R: A Language and Environment for Statistical Computing 2012 Vienna, Austria: R Foundation for Statistical Computing ISBN 3-900051-07-0. [http://www.R-project.org]

[B35] McMurrough TA Dickson RJ Thibert SMF Gloor GB Edgell DR Control of catalytic efficiency by a co-evolving network of catalytic and non-catalytic residues arXiv April 2014 [http://arxiv.org/abs/1404.3917] 10.1073/pnas.1322352111 PMC4060692 24912189

[B36] Soneson C Delorenzi M A comparison of methods for differential expression analysis of RNA-seq data BMC Bioinformatics 2013 14 91 10.1186/1471-2105-14-91 23497356 PMC3608160

[B37] Bottomly D Walter NAR Hunter JE Darakjian P Kawane S Buck KJ Searles RP Mooney M McWeeney SK Hitzemann R Evaluating gene expression in C57BL/6J and DBA/2J mouse striatum using RNA-seq and microarrays PLoS One 2011 6 3 17820 10.1371/journal.pone.0017820 PMC3063777 21455293

[B38] Frazee AC Langmead B Leek JT Recount: a multi-experiment resource of analysis-ready RNA-seq gene count datasets BMC Bioinformatics 2011 12 449 10.1186/1471-2105-12-449 22087737 PMC3229291

[B39] Scott M Gunderson CW Mateescu EM Zhang Z Hwa T Interdependence of cell growth and gene expression: origins and consequences Science 2010 330 6007 1099 102 10.1126/science.1192588 21097934

[B40] Altman DG Bland JM Measurement in medicine: the analysis of method comparison studies J R Stat Soc Series D (Statistician) 1983 32 3 307 17

[B41] HMQCP – QIIME Community Profiling [http://downloads.hmpdacc.org/data/HMQCP/otu_table_psn_v13.txt.gz] Accessed 1 Ju 2010

[B42] Segata N Haake SK Mannon P Lemon KP Waldron L Gevers D Huttenhower C Izard J Composition of the adult digestive tract bacterial microbiome based on seven mouth surfaces, tonsils, throat and stool samples Genome Biol 2012 13 6 42 10.1186/gb-2012-13-6-r42 PMC3446314 22698087

[B43] Legendre P Gallagher ED Ecologically meaningful transformations for ordination of species data Oecologia 2001 129 2 271 80 10.1007/s004420100716 28547606

[B44] Dixon P VEGAN, a package of R functions for community ecology J Vegetation Sci 2003 14 6 927 30 10.1111/j.1654-1103.2003.tb02228.x

[B45] Tarazona S García-Alcalde F Dopazo J Ferrer A Conesa A Differential expression in RNA-seq: a matter of depth Genome Res 2011 21 12 2213 23 10.1101/gr.124321.111 21903743 PMC3227109

[B46] Liu Y Zhou J White KP RNA-seq differential expression studies: more sequence or more replication? Bioinformatics 2013 30 3 301 4 24319002 10.1093/bioinformatics/btt688 PMC3904521

[B47] Auer PL Doerge RW Statistical design and analysis of RNA sequencing data Genetics 2010 185 2 405 16 10.1534/genetics.110.114983 20439781 PMC2881125

